# Health and social policies to advance Brazil’s End TB agenda during and after COVID-19: An analysis from tripartite governance and normative innovation perspectives

**DOI:** 10.1371/journal.pone.0345867

**Published:** 2026-04-03

**Authors:** Natacha Martins Ribeiro, Mônica Cristina Ribeiro Alexandre d’Auria de Lima, Aline Aparecida Monroe, André Luiz Teixeira Vinci, Beatriz Fornaziero Vigato, Rebeca Sousa Braga, Melisane Regina Lima Ferreira, Valter Chicalo António Caripa, Rander Junior Rosa, Nathalia Zini, Heriederson Sávio Dias Moura, Willie Otávio Bueno Bernardi, Jaqueline Garcia de Almeida Ballestero, Pedro Fredemir Palha, Rubia Laine de Paula Andrade, Ricardo Alexandre Arcêncio

**Affiliations:** Department of Maternal-Infant Nursing and Public Health, University of São Paulo at Ribeirão Preto College of Nursing, Ribeirão Preto, São Paulo, Brazil; Public Library of Science, UNITED KINGDOM OF GREAT BRITAIN AND NORTHERN IRELAND

## Abstract

**Objectives:**

To critically analyze, through a systematic documentary approach, the health and social policies implemented in Brazil to address tuberculosis during and after the COVID-19 pandemic, identifying advances, challenges, and gaps in federal coordination.

**Methods:**

Qualitative documentary study of 438 normative documents published between 2020 and 2024, including laws, decrees, ordinances, and resolutions at the federal, state, and municipal levels. Thematic content analysis was applied to examine the empirical material.

**Results:**

Six thematic categories emerged: financial aspects and resources; management and policies; social support; social context and community participation; development and innovation; and health care strategies. The analysis revealed lack of coordination among governmental spheres, regional inequalities, and gaps in innovation and management.

**Conclusion:**

Addressing TB in the post-pandemic context requires federal integration, strengthened evidence-based policies, and investments in innovation, offering relevant lessons for countries with similar socioeconomic contexts.

## Introduction

The COVID‑19 pandemic profoundly disrupted health systems worldwide, including in Brazil, redirecting resources to the emergency response and reducing diagnosis, treatment, and prevention of other conditions such as tuberculosis (TB) [1], which remains a major global public health challenge.

The socioeconomic consequences of the pandemic, intensified by cuts in social protection, deepened structural inequalities and weakened intersectoral responses to the social determinants of TB [2–4]. In Brazil, the Unified Health System (SUS) is a universal, free, tripartite system that depends on coordinated action among federal, state, and municipal levels to ensure comprehensive and equitable care.

During the pandemic, however, policies to confront TB were fragmented and poorly articulated, exposing persistent weaknesses in federal coordination, compromising continuity of care, and contributing to the serious TB situation observed in the following years [5–7], with elevated TB incidence (40.0 cases per 100,000 inhabitants), mortality (2.1 deaths per 100,000), and treatment abandonment (12.9%) in 2024 [8]

Brazil reaffirmed its global commitment to End TB at the United Nations High‑Level Meeting on Tuberculosis, held in partnership with the World Health Organization, and more recently launched the Healthy Brazil Program, which aims to expand prevention, integrate care for vulnerable groups, strengthen support systems, and foster research and innovation in TB care [7].

In the post‑pandemic period, multiple initiatives have emerged to advance the TB agenda, yet few studies have critically examined these policies, their implementation, and their real contribution to accelerating progress toward TB elimination in the country. Most studies on TB and COVID‑19 have focused on epidemiological trends and service disruptions, with little attention to how health and social policies were normatively constructed, implemented, and aligned across levels of government [8–13]. The specific gap this study addresses is the absence of a systematic, nationwide analysis of TB‑related norms and programs that explicitly examines two key dimensions of governance—federal integration and regulatory innovation—during and after the pandemic.

According to literature, normative innovation refers to the creation, revision, or adaptation of regulatory instruments that introduce novel mechanisms to address emerging health challenges, distinct from routine regulation which merely reiterates existing protocols (GARNEY et al., 2022).

Then, operational indicators include: (1) integration of digital technologies (e.g., vDOT for TB monitoring); (2) intersectoral coordination across federal spheres; (3) evidence-based adjustments to epidemiological realities; and (4) expansion of access through new legal frameworks for vulnerable populations. This study operationalizes these indicators to assess TB policy evolution during and post-COVID-19 [12]

By applying these concepts to a systematic documentary analysis of health and social policies for TB in Brazil, this study crosses the current frontier of knowledge by moving beyond descriptive epidemiology to reveal how federal coordination and innovative regulatory strategies—or their absence—have shaped the TB response during and after COVID‑19.

Therefore, the study aimed to critically analyze, through a systematic documentary approach, the health and social policies implemented in Brazil to combat TB during and after the COVID-19 pandemic, identifying advances, challenges, and gaps in federal coordination.

## Materials and methods

### Design

Qualitative documentary study [13,14], grounded in the conceptual framework on federative integration and normative innovation [15–16]. Documentary research is characterized by the search for information in original documents without prior analytical treatment [11,17], enabling the sociocultural and historical contextualization of the study object and facilitating its understanding [11].

### Data source

A total of 438 normative documents (laws, decrees, ordinances, resolutions) published between March 2020 and December 2024 were collected from the official websites of the Ministry of Health, the Federal Official Gazette, and state and municipal portals. National and subnational documents directly related to TB were included.

### Inclusion and exclusion criteria

Documents published between March 2020 and December 2024 (the COVID-19 pandemic and post-pandemic periods) were included. It is noteworthy that the pandemic period began in March 2020 and ended in May 2023 [18–19]. Eligibility criteria encompassed federal, state, and municipal laws; ministerial and secretarial ordinances; executive decrees; technical regulations; and other official documents. Only documents written in Portuguese were considered. Documents issued by international organizations were excluded, as were those related to military retirement due to tuberculosis and those concerning worker dismissal due to travel.

### Search and selection of documents

The searches were conducted in the following official national public portals: Ministry of Health website (https://www.gov.br/saude/pt-br/centrais-de-conteudo/publicacoes), Federal and State Official Gazette (DOU) (https://www.in.gov.br/consulta/), the Brazilian Federal Legislation Portal (https://legislacao.presidencia.gov.br/) for federal documents, and the State Laws Portal (https://leisestaduais.com.br/) for state and municipal documents. The search used only the keyword “tuberculosis,” without Boolean operators or additional terms. All analyzed documents were officially available in digital format, as normative acts in Brazil are required to be published online.

Document selection was conducted independently by three researchers. Google Sheets was used to store and manage the content and documentation, considering the type of each document, year of publication, issuing body or institution, and topics addressed. Duplicates were manually removed during the selection process by verifying document number, title, and publication date. In line with open science practices, all supplementary material related to the study will be made publicly available.

### Data analysis

The documents selected during data collection were saved in PDF format and transferred to Atlas.ti® software, version 23, for data organization and interpretation.

In this software, the selected documents were distributed into folders corresponding to each state and one to the federal government. Next, the analysis of the documents began with thematic content analysis, according to Bardin’s assumptions [20]: document pre-analysis, exploration of the material, and treatment of the results.

In the pre-analysis stage, a cursory reading of the documents was carried out in order to assess their relevance to the purposes of the study. To this end, five analytical dimensions were considered: context, interests at stake, reliability of sources, authorship, and key concepts present in the texts, as suggested by Cellard (2012) [21]. Based on the exploration of the selected materials, the analytical corpus of the study was constituted. From this corpus, thematic categories emerged, which served as the basis for the identification and organization of the units of meaning.

The development of thematic categories followed an inductive approach grounded in the principles of content analysis. Subsequently, open coding was conducted in Atlas.ti, in which textual segments were identified and grouped according to similarity.

The analytical process was iterative, allowing for constant comparison among codes, their refinement, and reorganization until the thematic categories were consolidated. Thematic saturation was considered reached when further readings no longer generated new codes.

The analytical consistency and coherence of the categories were ensured by three independent reviewers, who assessed the codes and discussed divergences in consensus meetings.

The volume of citations for each thematic category within the included documents was illustrated using a Sankey diagram, which depicts the density of the publications identified. The thickness of the connections varied, indicating which categories had a greater volume of information or associated documents. The color transition from green to blue represented the flow from the thematic categories to the final node, allowing for a clear visualization of each category’s relative contribution to the overall dataset.

### Ethical aspects

The research was approved by the Research Ethics Committee of the University of São Paulo at Ribeirão Preto College of Nursing, Brazil, under approval number 6,883,358 (CAAE: 77420524.1.0000.5393), on July 12, 2024. Informed consent was waived because the research involved the analysis of publicly available documents.

## Results

### Documentary characterization

Of the 438 documents, 318 (72.6%) were laws, 81 (18.5%) were decrees, 28 (6.4%) were ordinances, 6 (1.4%) were resolutions, 4 (0.9%) were public consultations, and 1 (0.2%) was a recommendation.

### Emerging thematic categories

From the processing of the empirical material, six thematic categories emerged (Table 1): Financial Aspects and Resources; Management and Policies; Social Support; Social Context and Community Participation; Development and Innovation; and Health Care Strategies. The units of meaning for each thematic category and their main findings are presented in Table 1.

**Table 1:** Mapping of Social and Health Policies on Tuberculosis in Brazil from 2020 to 2024: Analysis of Municipal, State, and.

Thematic Categories	Locations	Units of Meaning	Main Findings
*Financial Aspects and Resources* *n = 54 (12.33%)*	Rio grande do Sul (35; 64,81%) Federal (10; 18,52%) São Paulo (05; 9,26%) Minas Gerais (04; 7,71%)	Credit Allocation for TB	Allocation of federal and state resources to strengthen TB surveillance actions during the COVID-19 pandemic, ensuring continuity of diagnosis and treatment. [22]
		Credit Allocation for Epidemiological Surveillance	Authorization of extraordinary credits for epidemiological surveillance, with emphasis on tackling TB in vulnerable populations, reinforcing the continuity of control programs even during health emergencies. [23]
		Equipment Acquisition	Allocation of resources for the acquisition of equipment aimed at TB diagnosis, strengthening the healthcare network, and modernization of health units. [24]
*Social Context and Community Participation* *n = 16 (3,65%)*	Federal (08; 50%) Minas Gerais (04; 25%) Espírito Santo (01; 6,25%) Mato Grosso (01; 6,25%) Rio de Janeiro (01; 6,25%) Rio Grande do Sul (01; 6,25%)	Civil Society Actions	Encouragement of community participation in the planning and execution of tuberculosis control actions, strengthening the integration between civil society and health services. [24]
		Social Determinants of Tuberculosis	Inclusion of the social determinants of health approach in TB control, with intersectoral strategies that consider factors such as poverty, housing, and access to services. [25]
		Care Recommendations	Definition of comprehensive care guidelines for people affected by tuberculosis, with emphasis on treatment continuity and multiprofessional follow-up [8]
*Development and Innovation* *n = 4 (0,91%)*	Alagoas (01; 25%) Amazonas (01; 25%) Federal (01; 25%) Mato Grosso (01; 25%)	Professional Training	Promotion of training actions for health professionals in the context of tuberculosis, focusing on updating clinical protocols and best care practices. [26]
		Communication Strategies	Encouragement of qualified communication and the use of digital tools to guide the population and health professionals on tuberculosis prevention and treatment. [26]
		Digital Health	
		Rifampicin Distribution	Expansion of rifampicin and other essential drug distribution to ensure continuity of TB treatment during the pandemic. [27]
*Healthcare Strategies* *n = 63 (14,38%)*	Federal (22; 34;92) Minas Gerais (13; 20,36%) Mato Grosso do Sul (05; 7;94) Rio Grande do Sul (05; 7,94%) Sergipe (04; 6,35%) Amazonas (02; 3,17%) Bahia (02; 3,17%) Santa Catarina (02; 3,17%) Alagoas (01; 1,59%) Amapá (01; 1,59%) Ceará (01; 1,59%) Mato Grosso (01; 1,59%) Pará (01; 1,59%) Pernambuco (01; 1,59%) Rio de Janeiro (01; 1,59%) São Paulo (01; 1,59%)	Screening and Active Case-Finding Strategies	Implementation of active case-finding strategies for respiratory symptomatic individuals and contact tracing of TB cases to control community transmission. [27]
		Medication Strategies	Update of tuberculosis drug treatment protocols to expand access and ensure treatment adherence. [27]
		Diagnostic Strategies	Expansion of TB diagnostic capacity, including rapid tests and strengthening the supporting laboratory network. [28]
		Strategies for Vulnerable Populations	Implementation of specific actions aimed at populations in situations of social vulnerability, such as Indigenous peoples, homeless individuals, and incarcerated persons. [29]
		Prevention	Intensification of tuberculosis prevention strategies, with emphasis on educational campaigns and active surveillance actions. [29]
		BCG Vaccine	Maintenance of BCG vaccination as a fundamental strategy for preventing childhood tuberculosis. [28]
*Management and Policies* *n = 27 (6,16%)*	Federal (12; 44,4%) Amazonas (03; 11,11%) Minas Gerais (03; 11,11%) Rio Grande do Sul (03; 11,11%) Mato Grosso do Sul (02; 11,11%) Rio de Janeiro (02; 7,41%) Alagoas (01; 3,70) Mato Grosso (01; 3,70)	Network Expansion	Definition of strategies to expand the network of primary and specialized care for tuberculosis management. [29]
		Government Commitment	Strengthening of institutional commitment to eliminate tuberculosis as a public health problem, formalized through intergovernmental agreements. [30]
		Intersectorality in TB Control	Establishment of coordinated actions among health, social assistance, and other sectors for comprehensive TB control. [25]
		National Policies for TB Elimination	Formulation of public policies with specific targets for tuberculosis elimination by 2035. [25]
*Social Support* *n = 274 (62%)*	Rio Grande do Sul **(104; 37,94%)** Paraná **(42; 15,33%)** São Paulo **(32; 11,68%)** Santa Catarina **(26; 9,49%)** Mato Grosso **(20; 7,30%)** Minas Gerais **(12; 4,38%)** Bahia **(07; 2,55%)** Goiás **(06; 2,19%)** Rondônia **(06; 2,19%)** Federal **(03; 1,09%)** Pará **(03; 1,09%)** Pernambuco **(03; 1,09%)** Paraíba **(02; 0,73%)** Rio de Janeiro **(02; 0,73%)** Alagoas **(01; 0,36%)** Espírito Santo **(01; 0,36%)** Rio Grande do Norte **(01; 0,36%)** Roraima **(01; 0,36%)** Sergipe **(01; 0,36%)** Tocantins **(01; 0,36%)**	Food Assistance	Establishment of food security programs for people affected by tuberculosis, aiming to reduce treatment abandonment due to food insecurity. [31]
		Benefits and Social Support	Granting of social benefits and assistance to vulnerable populations affected by tuberculosis in the context of the pandemic. [32]
		Fee Exemption	Fee exemptions for vulnerable populations aimed at promoting greater access to health services and social rights. [32]
		Social Security	Inclusion of social security guarantees and social protection for people affected by tuberculosis within the scope of state and municipal programs. [32]
		Income Transfer Programs	Expansion of income transfer programs for families affected by TB, aiming to mitigate the economic impact of the disease. [32]

**Fig 1:**
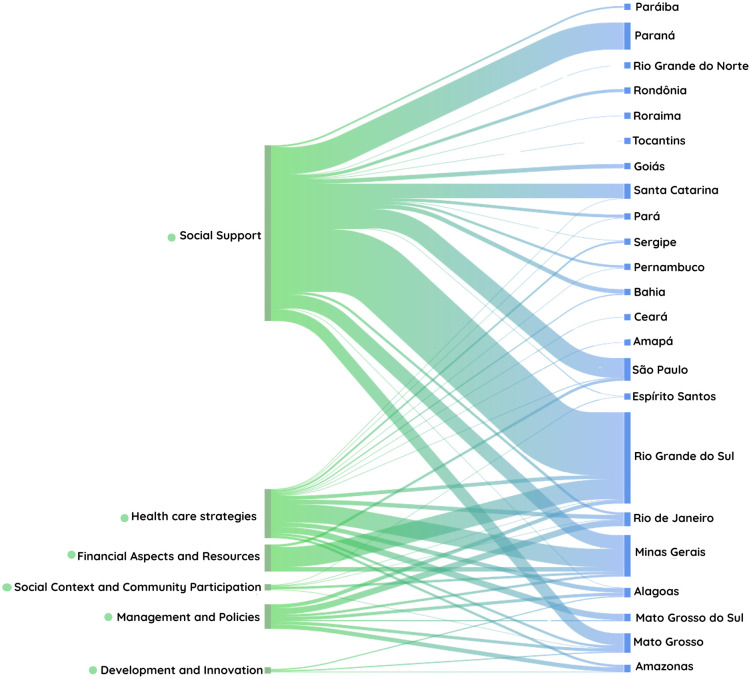
The color coding indicates the six thematic axes identified in the documentary analysis, enabling a clear visualization of how each category is distributed across the federative units. The thickness of each flow represents the volume of documents within that thematic category, while gradual color shifts within the flows reflect transitions between categories and the relative intensity of connections across states.

The Sankey diagram showed that **“Social Support”** was the most frequently addressed thematic category in state and municipal documents, with Rio Grande do Sul leading in the creation of policies in this area, followed by Paraná, São Paulo, Santa Catarina, Minas Gerais, and Mato Grosso. Following social support, the category **“TB Care Strategies”** stood out, appearing most often in documents from municipalities in Minas Gerais. In the category **“Financial Aspects and Resources,”** Rio Grande do Sul had the highest number of documents. In the category **“Management and Policies,”** no state stood out compared to the others. The same pattern was observed for the less frequently addressed categories: **“Social Context and Community Participation”** and **“Development and Innovation”** (Fig 1).

**Fig 2:**
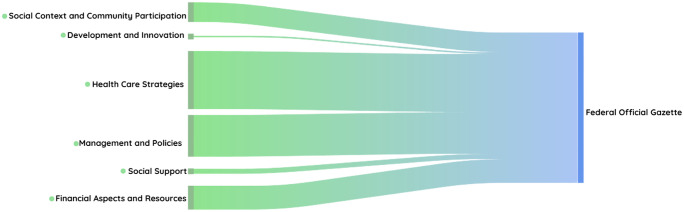
The color gradient represents the thematic axes identified in the analysis: green tones correspond to the different categories of TB-related actions and policies, while the transition to blue indicates the destination of these documents in the Federal Official Gazette. The thickness of each flow reflects the volume of documents associated with each thematic category.

With regard to the federal documents, it was observed that the highest weights of documentation were related to “health care strategies”, followed by “management and policies”, “financial aspects and resources” and “social context and community participation”. “Social support” and “development and innovation” were the least addressed categories (Fig 2)

## Discussion

The study aimed to critically analyze, through a systematic documentary approach, the health and social policies implemented in Brazil to address TB during and after the COVID-19 pandemic, identifying advances, challenges, and gaps in federal coordination.

There is evidence of normative fragmentation, indicating failures in coordination among the federal government, states, and municipalities—failures that were exacerbated during the pandemic by divergent political positions and the absence of centralized leadership. Another relevant finding is that, despite advances in social support and health care, critical gaps remain in technological innovation, capacity building, and management, which limit the effectiveness of policies. These gaps may be related to the limited installed capacity in some states to incorporate digital technologies during the pandemic, the lack of specific national guidelines for technological integration in the TB response [14,22,33,34], and longstanding inequalities in digital infrastructure and professional training across federative units.

The heterogeneity observed in normative production may also be related to socioeconomic and institutional factors that vary substantially across Brazilian states. Federative units with higher Gross Domestic Product (GDP), lower social inequality, and greater administrative capacity tend to have more consolidated management structures, more stable technical teams, and greater availability of resources to formulate and revise regulations, especially in crisis contexts [6,35]. In addition, states that already had more structured tuberculosis control programs in the pre-pandemic period may have responded more rapidly to emerging regulatory demands [1,12]. Conversely, states with more unfavorable socioeconomic indicators—such as higher informality, lower fiscal capacity, and lower administrative professionalization—face technical and operational limitations that may restrict their normative capacity, contributing to the asymmetry observed in the country [2,4]. These inequalities reflect historical challenges in Brazilian federative governance and help explain why some states produced more TB-related regulations than others during and after the pandemic [8].

The findings reinforce the need for coordinated action among federal, state, and municipal levels to ensure the allocation of financial resources aligned with the guidelines of the National Plan to End TB [8] and the Recommendations Plan [36], which emphasize the division of roles among federative entities: the federal government as regulator and financier; states as regional coordinators; and municipalities as direct implementers of actions. Thus, this study offers an innovative contribution by mapping and comparing the normative production of these entities.

The substantial normative output related to social support, particularly by municipalities and states, indicates a locally driven prioritization in response to the health emergency. This movement may be linked to the national political context during the pandemic, marked by denialist positions and the spread of misinformation, which required more immediate and autonomous responses from subnational governments to mitigate the social impacts of the crisis [37]. Such measures had a direct positive impact on individuals and families affected by TB, as the disease is a marker of health inequities and contributes to widening social disparities due to catastrophic costs [35,38,39].

Fewer policies were created by the federal government, with a large volume of documents originating from municipalities and/or states—particularly Rio Grande do Sul, Paraná, and São Paulo. These states have higher Gross Domestic Product (GDP) and lower Gini indices, meaning they are wealthier and have less income inequality. This suggests the hypothesis that these states sought their own mechanisms to address TB during the critical period, while states with poorer social indicators may have had fewer conditions to implement such policies. This reinforces issues of regional inequality, running counter to the SUS principle of equity, and poses a challenge to achieving the goals of the End TB strategy.

Moreover, the discrepancy between state records and federal government publications suggests a possible misalignment between local demands and national priorities, highlighting the need for greater responsiveness from the federal government [40]. In this regard, it would be necessary for the federal government to direct greater attention to these states to prevent the deepening of poverty and inequality through equitable investments, ensuring that all federative units have the means to provide effective and inclusive social support. This would represent a form of normative innovation, aimed at improving regulatory frameworks, developing new legal instruments, and continuously updating rules to promote sustainability, efficiency, and expanded access—especially in strategic areas such as health.

Even with the existence of federal documents aimed at strengthening health care strategies, not all states developed regulations to expand such strategies. During the pandemic, the federal government’s response—supported by a robust legal framework reinforced by some states and municipalities—demonstrated the country’s capacity to sustain the formulation and implementation of public health policies to address TB.

Studies show that several countries faced serious challenges during the pandemic in maintaining TB control, particularly regarding medication distribution, laboratory support, and diagnostics [25,41,42]. Brazil followed a similar trend, revealing comparable structural challenges. However, the regulations and agreements established by federal and state governments during and after the pandemic indicate recognition of the need to strengthen and resume efforts to confront the disease, reinforcing institutional commitment to the continuity and improvement of TB control policies.

The heterogeneity in the regulation of financial aspects across some states may have hindered the equitable distribution of resources, as national regulations—although relevant—do not account for state and municipal specificities, thereby deepening internal and regional inequalities [43]. This heterogeneity underscores the need to strengthen governance and intersectoral coordination to ensure sustainable financing that is aligned with local needs and global strategies for TB elimination.

In this regard, recognizing and prioritizing cost-effective strategies aimed at affected populations is essential for the appropriate allocation of resources. Directly Observed Therapy (DOT) is considered the primary cost-effective strategy for TB treatment; however, its national coverage is only 38.9% among individuals undergoing treatment [44].

Regarding management and policies, municipalities appear to be aligning their actions with federal guidelines, as they produced few regulations in this area. In this context, the role of the State was fundamental in providing direction, establishing criteria for expanding the care network, and fostering the commitment of municipalities and states in combating TB and promoting intersectoral coordination, given the multiple determinants of the disease.

The prioritization of certain policies over others by municipalities and states is concerning, especially in regions with high TB incidence, such as Rio de Janeiro and Amazonas. Although these states introduced laws for the management and control of the disease, their implementation may have been hindered during the pandemic due to the lack of guidelines on the procurement of resources and equipment for TB control [42].

In the analysis of social context and community participation, municipalities and states with a strong tradition of social mobilization—such as Minas Gerais and Rio de Janeiro—tended to demonstrate greater community engagement and civil society involvement in the formulation and implementation of public policies in this area [3]. This engagement has been crucial for the success of prevention and care strategies, especially among socially vulnerable populations such as people experiencing homelessness, Indigenous peoples, and individuals deprived of liberty [45–47], as well as for reducing health inequities and promoting a more equitable health system.

The low volume of normative production regarding development and innovation may be associated with the absence of consistent national guidelines for digital integration in TB care, disparities in technological infrastructure across states and municipalities, and the challenges of reorganizing services during the pandemic [14,33,34,48].

However, within this thematic category, it is important to highlight the strategy of video-Observed Therapy (vDOT), which was essential for monitoring people with TB during the pandemic and was formalized through Informative Note No. 20/2023. Studies indicate that digital resources such as recordings and video calls are fundamental for monitoring treatment, especially in contexts of social isolation [14]. Promising results have been observed with this strategy [33–34]. It has been incorporated into policies in several countries [48], and in Brazil—where TB continues to show high incidence and mortality rates [10]—there is a clear need for investments that expand and strengthen digital tools.

The absence of more robust regulations contrasts with countries that have adopted digital health strategies in a systematic and coordinated manner. This underscores the urgency of structural investments in innovation and technological capacity in Brazil, especially for monitoring chronic and infectious diseases during health crises [19,48].

These findings reveal the need to strengthen federative governance mechanisms in Brazil. For federal managers, recommendations include strengthening tripartite coordination, defining national guidelines for normative responses during health emergencies, and ensuring continuous investment in digital infrastructure and professional training. For state managers, it is essential to consolidate permanent technical teams, improve administrative capacity, and develop regulatory governance units capable of responding quickly to changes in the epidemiological landscape. For municipal managers, recommendations include expanding active case-finding strategies, strengthening territorial surveillance actions, and increasing the use of digital tools such as VDOT.

This study may contribute to countries with geopolitical and socioeconomic contexts similar to Brazil’s by highlighting the importance of a stronger federal presence in TB management. The documentary analysis presented offers robust evidence and allows us to infer that, despite advances in some areas—such as social support and care strategies—critical regulatory gaps remain in areas such as innovation and governance. The unequal distribution of regulations across levels of government and regions reflects failures in federative coordination and an insufficient response to local demands, further exacerbated by the political context of the pandemic.

Compared with international experiences, it becomes evident that Brazil must strengthen the integration of evidence-based public policies and promote greater equity in the regulation and implementation of actions. This study contributes by revealing patterns that remain underexplored in the literature and may offer insights for other nations regarding the importance of coordination across different levels of government.

## Limitations

A limitation of the study is that it focuses solely on the analysis of regulations (normative content), without a direct evaluation of the implementation or the impact of the policies on health outcomes—an aspect that would be valuable to consider in future research. Additionally, the study is based on the collection of official documents; however, since the documents were obtained through an online search process, some non-digital materials may not have been included. Furthermore, the selection of materials was focused on tuberculosis, so some policies that may have contributed indirectly to TB control—such as emergency financial aid—might not have been included.

However, this study advances knowledge by highlighting the Brazilian pattern of tuberculosis (TB) response during a crisis period intensified by the COVID-19 pandemic. It reveals challenges that may be shared by countries with large territorial dimensions and socioeconomic inequalities, offering lessons for multilevel health governance through tripartite participation and management. The policies formulated thus far converge in their common goal of promoting broader access to health and social protection actions for the population, as well as greater management capacity for different levels of government, aiming to increase the effectiveness of these actions. It should be noted that federative integration must be strategic to ensure the universality, equity, and comprehensiveness of access to health, thereby advancing the agenda for TB elimination.

Normative innovation brings the prerogative of adapting policies through the creation, revision, and implementation of rules and laws that seek to respond creatively and efficiently to the challenges of the Unified Health System (SUS), incorporating scientific, technological, and social advances. However, this process must be orchestrated within the tripartite structure, which was identified in the study as a point of fragility.
